# Pyroptosis-Related LncRNA Signature Predicts Prognosis and Is Associated With Immune Infiltration in Hepatocellular Carcinoma

**DOI:** 10.3389/fonc.2022.794034

**Published:** 2022-03-03

**Authors:** Ze-Kun Liu, Ke-Fei Wu, Ren-Yu Zhang, Ling-Min Kong, Run-Ze Shang, Jian-Jun Lv, Can Li, Meng Lu, Yu-Le Yong, Cong Zhang, Nai-Shan Zheng, Yan-Hong Li, Zhi-Nan Chen, Huijie Bian, Ding Wei

**Affiliations:** ^1^National Translational Science Center for Molecular Medicine, Department of Cell Biology, State Key Laboratory of Cancer Biology, Fourth Military Medical University, Xi’an, China; ^2^Department of General Surgery, Affiliated Haixia Hospital of Huaqiao University (The 910 Hospital of the Joint Logistics Team), Quanzhou, China; ^3^Department of Gynaecology and Obstetrics, Tangdu Hospital, Fourth Military Medical University, Xi’an, China

**Keywords:** hepatocellular carcinoma, long non-coding RNA, pyroptosis, immune infiltration, immunotherapy

## Abstract

Pyroptosis is an inflammatory form of programmed cell death that is involved in various cancers, including hepatocellular carcinoma (HCC). Long non-coding RNAs (lncRNAs) were recently verified as crucial mediators in the regulation of pyroptosis. However, the role of pyroptosis-related lncRNAs in HCC and their associations with prognosis have not been reported. In this study, we constructed a prognostic signature based on pyroptosis-related differentially expressed lncRNAs in HCC. A co-expression network of pyroptosis-related mRNAs–lncRNAs was constructed based on HCC data from The Cancer Genome Atlas. Cox regression analyses were performed to construct a pyroptosis-related lncRNA signature (PRlncSig) in a training cohort, which was subsequently validated in a testing cohort and a combination of the two cohorts. Kaplan–Meier analyses revealed that patients in the high-risk group had poorer survival times. Receiver operating characteristic curve and principal component analyses further verified the accuracy of the PRlncSig model. Besides, the external cohort validation confirmed the robustness of PRlncSig. Furthermore, a nomogram based on the PRlncSig score and clinical characteristics was established and shown to have robust prediction ability. In addition, gene set enrichment analysis revealed that the RNA degradation, the cell cycle, the WNT signaling pathway, and numerous immune processes were significantly enriched in the high-risk group compared to the low-risk group. Moreover, the immune cell subpopulations, the expression of immune checkpoint genes, and response to chemotherapy and immunotherapy differed significantly between the high- and low-risk groups. Finally, the expression levels of the five lncRNAs in the signature were validated by quantitative real-time PCR. In summary, our PRlncSig model shows significant predictive value with respect to prognosis of HCC patients and could provide clinical guidance for individualized immunotherapy.

## Introduction

Hepatocellular carcinoma (HCC) is a common malignancy, with a high incidence-to-mortality ratio and most cases detected at late stages (1). Chronic hepatitis B and hepatitis C, alcohol addiction, aflatoxins, and metabolic liver disease are important risk factors for HCC (2). Although various treatments are available, including surgery, radiotherapy, and chemotherapy, patients with advanced HCC have poor prognosis, with 5-year survival rates of around 15% (3). The intra-tumoral heterogenicity of HCC limits the accuracy and applicability of current prediction and diagnosis methods (4). Therefore, it is particularly important to develop a reliable evaluation model with improved prediction efficiency for the prognosis of HCC.

Pyroptosis is a form of programmed cell death initiated by inflammasomes, and its mechanism involves the caspase-mediated cleavage of gasdermins, which leads to the formation of pores in the plasma membrane, followed by release of inflammatory factors and, ultimately, ruptures of cell membrane and death of the cell (5). Recent studies have shown that pyroptosis may have a dual role in tumor pathogenesis. Gasdermin E was shown to be a tumor suppressor that can suppress colony formation and cell proliferation in gastric cancer, melanoma, and colorectal cancer, as well as cell invasion in breast cancer (6). Moreover, pyroptosis-induced inflammation was found to enhance the functional properties of tumor-infiltrating immune cells and induce strong anti-tumor immunity (6, 7). Normal tissues and cells were constantly stimulated by inflammatory factors released by the activation of pyroptosis, which increased the risk of cancer (8, 9). Nevertheless, the effects of pyroptosis on the prognosis of HCC patients have remained unclear.

Long non-coding RNAs (lncRNAs) are transcript RNA molecules of about 200 nucleotides in length, and they have no ability to encode proteins but regulate the expression of genes (10). LncRNAs have important roles in biological regulatory processes and their dysfunction contributes to pathological conditions, including cancer growth. Many recent studies have suggested that lncRNAs are crucial mediators of the development of HCC. For example, Lnc-UCID and MCM3AP-AS1 were identified as novel oncogenic lncRNAs that could promote cell growth in HCC (11, 12). Chen et al. (13) found that lncRNA SNHG7 could exert an oncogenic effect by inhibiting pyroptosis in HCC. However, there is little evidence regarding the clinical significance and biological functions of pyroptosis-related lncRNAs in HCC.

**Figure 1:**
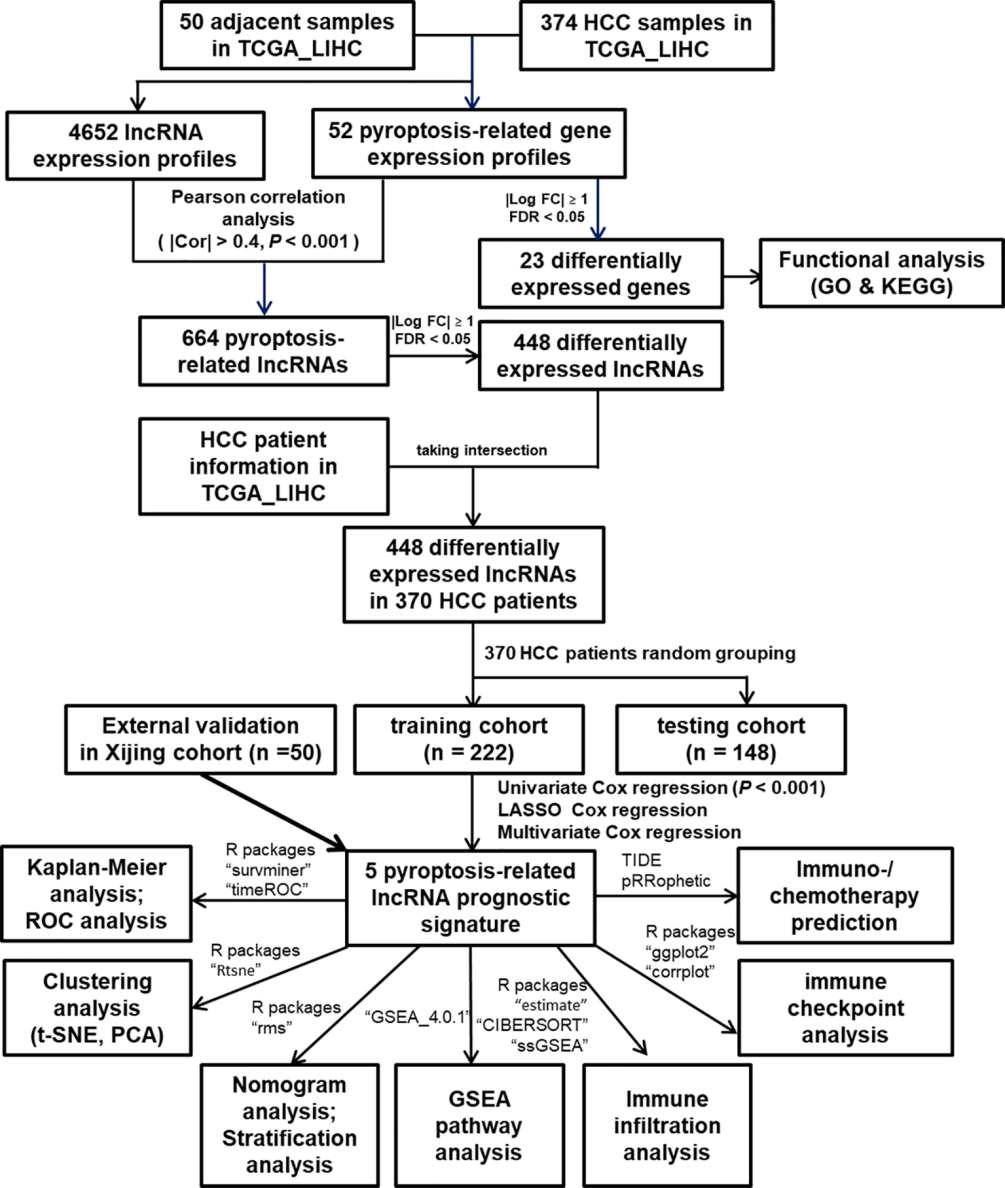
Flowchart of the study process.

In the present study, as shown in **Figure 1**, we built a novel pyroptosis-related lncRNA signature (PRlncSig) based on HCC data from The Cancer Genome Atlas (TCGA) database and systematically evaluated the prognostic significance of this signature and its relationship with clinicopathological characteristics of HCC patients. Moreover, we developed a nomogram to precisely predict the prognosis of HCC patients and improved the prediction efficiency for individuals. Finally, we explored the relationships between PRlncSig and immune cell infiltration and immunotherapeutic response. Our findings help to elucidate the regulatory mechanisms of pyroptosis in HCC, which may be conducive to improving prognostic evaluation and individual treatment for HCC patients.

## Materials and Methods

### HCC Patient Data and Processing

RNA sequencing (RNA-seq) data from HCC patient samples (including 374 liver tumor tissues and 50 adjacent tissues) and corresponding clinical characteristics were downloaded from TCGA (https://portal.gdc.cancer.gov/). The transcriptional profiling data of the TCGA_LIHC dataset was transformed from FPKM (fragments per kilobase million) values into TPM (transcripts per million) values. The “limma” package (version 3.48.1) (14) in R (version 4.1.0) was used to identify differentially expressed genes (DEGs) with false discovery rate (FDR) < 0.05 and |log_2_ (fold change) | ≥ 1.0.

### Identification of the Pyroptosis-Related LncRNAs in HCC

A total of 52 pyroptosis-related genes were acquired from the Molecular Signatures Database (MSigDB, http://www.gsea-msigdb.org/gsea/msigdb) and recent studies (15, 16), and the genes are presented in **Supplementary Table 1**. Pearson correlation analysis was performed to determine the correlations between the expression levels of pyroptosis-related genes and those of lncRNAs to identify the pyroptosis-related lncRNAs. The criteria were |Pearson correlation coefficient| > 0.4 and *P* < 0.001.

### Random Grouping of Data

A total of 370 HCC samples were matched with corresponding patients for whom complete survival information was available. The 370 samples were randomly divided into two groups, a training cohort (60%) and a testing cohort (40%), using the “caret” package (version 6.0.88) in R. The clinical features of the HCC patients after grouping are shown in **Table 1**.

**Table 1:** Clinicopathological characteristics of the HCC patient in each cohort.

Clinicopathological Variables	Training cohort (n = 222)	Testing cohort (n = 148)	Entire TCGA cohort (n = 370)	*P* value
Age				
<= 65	142(63.96%)	90(60.81%)	232(62.7%)	0.6138
> 65	80(36.04%)	58(39.19%)	138(37.3%)	
Gender				
Female	71(31.98%)	50(33.78%)	121(32.7%)	0.8035
Male	151(68.02%)	98(66.22%)	249(67.3%)	
Grade				
G1	33(14.86%)	22(14.86%)	55(14.86%)	0.3999
G2	110(49.55%)	67(45.27%)	177(47.84%)	
G3	66(29.73%)	55(37.16%)	121(32.7%)	
G4	9(4.05%)	3(2.03%)	12(3.24%)	
Unknow	4(1.8%)	1(0.68%)	5(1.35%)	
Stage				
Stage I	106(47.75%)	65(43.92%)	171(46.22%)	0.9276
Stage II	52(23.42%)	33(22.3%)	85(22.97%)	
Stage III	49(22.07%)	36(24.32%)	85(22.97%)	
Stage IV	3(1.35%)	2(1.35%)	5(1.35%)	
Unknow	12(5.41%)	12(8.11%)	24(6.49%)	

### Construction of a Pyroptosis-Related LncRNA Prognostic Signature for HCC

We performed univariate Cox regression analysis (*P* < 0.001) to identify survival-related lncRNAs, and constructed a pyroptosis-related lncRNA signature (PRlncSig) for HCC using the least absolute shrinkage and selection operator (LASSO) regression model and multivariate Cox regression analyses in the training cohort. We then verified PRlncSig in the testing cohort and the entire cohort. The risk score for each HCC patient was calculated by the following formula: risk score = (coefficient lncRNA1 × expression of lncRNA1) + (coefficient lncRNA2 × expression of lncRNA2) + ⋯ + (coefficient lncRNAn × expression of lncRNAn).

Using the median risk score in the training cohort as the cutoff value, the HCC patients were divided into two groups, a high-risk group and a low-risk group. Then, risk scores were calculated for all HCC patients in the testing cohort and the entire cohort, after which they were divided into high- and low-risk groups based on the same cutoff value. Kaplan–Meier survival analyses were performed for both low- and high-risk groups in all three cohorts using the “survival” package (version 3.2.11). *P* < 0.05 was considered to indicate statistical significance.

Time-dependent receiver operating characteristic (ROC) curves were constructed to evaluate the predictive accuracy and specificity of the risk model and of different clinicopathological characteristics (age, gender, grade, and stage) using the “timeROC” package (version 0.4).

### Principal Components Analysis

Principal component analysis (PCA) was conducted to verify whether the final genes can separate HCC samples with different subtypes. Considering the individual differences in HCC patients, PCA algorithms were used to calculate a risk score for each sample in order to quantify the risk patterns. PCA and t-distributed stochastic neighbour embedding (t-SNE) were carried out to evaluate the ability of the risk score to cluster HCC patients using the “Rtsne” (version 0.15) and “ggplot2” (version 3.3.3) packages.

### Construction and Calibration of Nomogram

A nomogram based on the risk score and clinical characteristics (gender, grade, age, stage) was established using the “rms” (version 6.2.0) and “regplot” (version 1.1) packages, for prediction of 1-, 3-, and 5-year overall survival of HCC patients. Then, ROC and calibration analyses were performed to verify the prediction accuracy of the nomogram.

### Functional Gene Set Enrichment Analysis (GSEA)

GSEA software (version 4.0.3) was used to analyze the critical pathways and enrichment terms related to PRlncSig in the entire TCGA cohort. The “c2.cp.kegg.v7.4.symbols” and “c7.all.v7.4.symbols (immunologic signatures)” gene sets were selected for GSEA analysis, and FDR < 0.05 was considered to indicate statistical significance.

### Analysis of Tumor-Infiltrating Immune Cells

Based on PRlncSig, the TIMER, CIBERSORT, CIBERSORT-ABS, QUANTISEQ, MCPCOUNTER, XCELL, and EPIC algorithms (17) were implemented to estimate the immune infiltration levels in the high-risk and low-risk groups. Single-sample GSEA (ssGSEA) was used to analyze the differences in immune cells and pathways between the two groups using the “GSVA” package (version 1.38.2) (18). The relevant markers of immune cells and pathways are shown in **Supplementary Table 2**. The ESTIMATE algorithm was used to evaluate the degree of infiltration of tumor cells and normal cells in order to calculate EstimateScore, ImmuneScore, StromalScore, and TumorPurity (19). The prediction efficiency of immune checkpoint inhibitors may be related to the expression of immune-checkpoint-related genes. Thus, the relationships between immune checkpoint genes and the pyroptosis-related signature were analyzed to investigate the potential role of PRlncSig and pyroptosis-related lncRNAs in immunotherapy for HCC.

### Prediction of Immunotherapeutic Response

Potential response to immunotherapy of HCC patients was predicted using the Tumor Immune Dysfunction and Exclusion (TIDE) algorithm (20). The “pRRophetic” package (version 0.5) (21) was used to predict drug sensitivity by calculating the half-maximal inhibitory concentration (IC50) for each sample.

### Quantitative Real-Time PCR (qRT-PCR)

Twenty-four pairs of HCC and adjacent tissues and twenty-six additional cases of HCC tissues (total 50 cases of HCC tissues as the external validation cohort) were obtained from Xijing Hospital, Fourth Military Medical University of China. Our study was approved by the Ethical Committee and Institutional Review Board of the Fourth Military Medical University. RNA was extracted using a total RNA kit II (Omega, USA), and cDNA was synthesized using a PrimeScript™ RT reagent Kit (TaKaRa Biotechnology, Japan). The mRNA expression was assessed in 96-well plates using an Mx3005P Real-Time PCR system (Agilent Technologies, Germany) with a SYBR Premix ExTaq kit (TaKaRa Biotechnology, Japan). The qRT-PCR data were analyzed using the △△Ct method with GAPDH as the reference gene. The primer sequences are listed in **Supplementary Table 3**.

### Statistical Analysis

Statistical analysis was performed using R software (version 4.1.0). Overall survival analysis was performed using the Kaplan–Meier method and log-rank tests. Univariate and multivariate Cox regression analyses were performed to calculate the prognostic significance of lncRNAs in HCC patients. Pearson rank was used in the correlation analysis. The statistical significance of differences between independent groups was calculated using student’s *t*-test. The criterion for statistical significance was set to *P* < 0.05.

## Results

### Identification of Differentially Expressed Pyroptosis-Related Genes and Functional Enrichment Analysis

**Figure 2:**
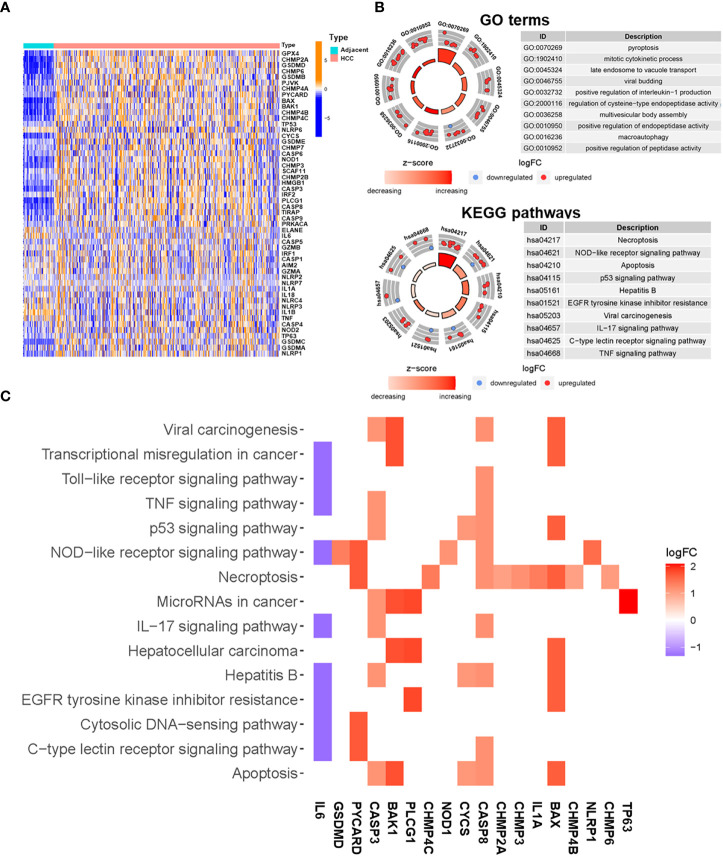
Functional enrichment analysis of differentially expressed pyroptosis-related genes. **(A)** Heatmap of expression of pyroptosis-related genes in HCC and adjacent tissues (blue: low expression level; dark orange: high expression level). **(B)** Enriched GO terms (up) and KEGG pathways (down) for differentially expressed pyroptosis-related genes. The outer circle shows a scatter diagram of the log fold change (FC) value allocated to each term. **(C)** Heatmap showing the relationships between differentially expressed pyroptosis-related genes and enriched KEGG pathways. Colors represent the logFC values of each gene in HCC compared with adjacent tissues.

The expression levels of 52 pyroptosis-related genes were compared using TCGA data from 374 HCC tumor tissues and 50 adjacent tissues. The mRNA expression profiles of these genes are presented in **Figure 2A**. A total of 23 DEGs that met the cut-off criteria were identified, of which one was downregulated and 22 were upregulated (**Supplementary Table 4**). Functional enrichment analysis was used to identify gene ontology (GO) terms and Kyoto Encyclopedia of Genes and Genomes (KEGG) pathways associated with the 23 DEGs. The following GO terms were significantly enriched in pyroptosis (*P* = 4.60E-17), mitotic cytokinetic process (*P* = 5.61E-11), late endosome to vacuole transport (*P* = 8.85E-17), and positive regulation of interleukin-1 production (1.16E-10) (**Figure 2B**). According to the KEGG pathway analysis, the DEGs were mainly involved in necroptosis (*P* = 1.43E-11), NOD-like receptor signaling pathway (*P* = 1.92E-06), p53 signaling pathway (*P* = 1.68E-05), and TNF signaling pathway (*P* = 1.80E-3) (**Figure 2B**). The relationships between DEGs and enriched pathways are presented in **Figure 2C**.

### Construction and Verification of PRlncSig for HCC

**Figure 3:**
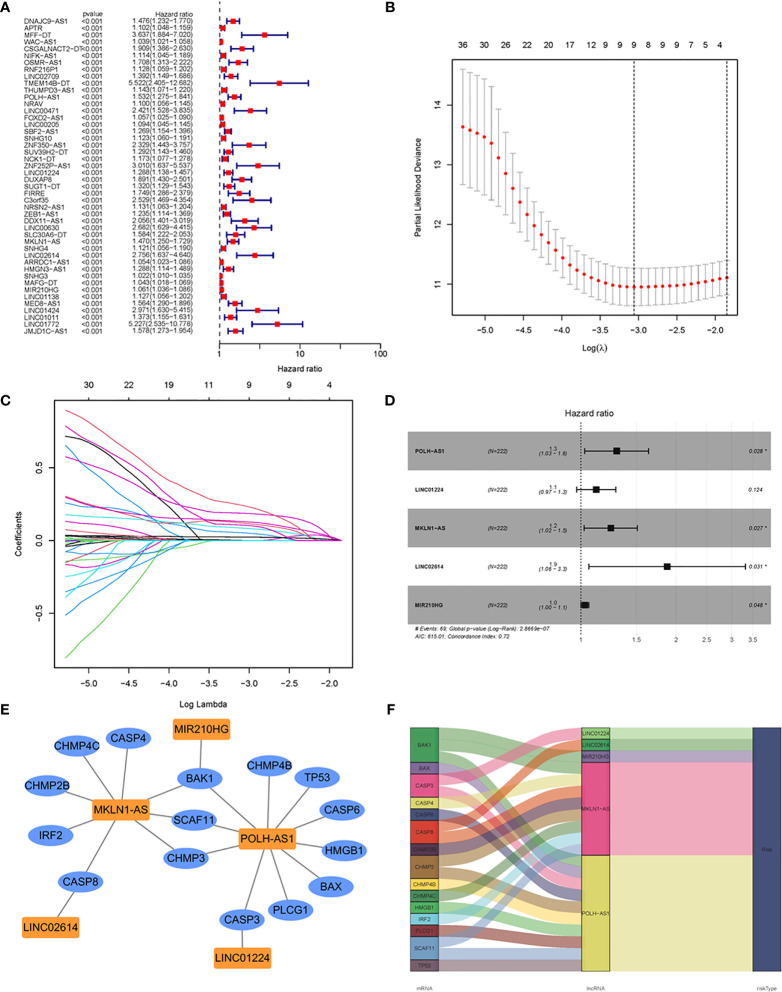
Construction of prognostic risk signature for patients with HCC based on pyroptosis-related lncRNAs in the training set. **(A)** Forest plot of 46 pyroptosis-related lncRNAs associated with overall survival of HCC patients based on univariate Cox regression analysis. **(B)** Distribution plot of the partial likelihood deviation of the LASSO regression. Nine variables were retained when the partial likelihood deviation reached the minimum (log lambda = –3.1). **(C)** Distribution plot of the LASSO coefficient. **(D)** Multivariate Cox regression analysis identified five pyroptosis-related lncRNAs for the construction of a prognostic model. **(E)** Prognostic co-expression network of the five pyroptosis-related lncRNAs and mRNAs. **(F)** Sankey diagram of the relationship between lncRNAs and mRNAs. *P < 0.05.

Using Pearson correlation analysis (|R^2^| > 0.4, *P* < 0.001), 664 pyroptosis-related lncRNAs were identified in HCC (**Supplementary Table 5**), of which 448 were considered to be differentially expressed (**Supplementary Table 4**). To explore the clinical significance of PRlncSig, 370 HCC samples were randomly divided into two cohorts, a training cohort (60%, n = 222 cases) and a testing cohort (40%, n = 148 cases). Subsequently, we performed univariate Cox regression analysis to screen the prognostic PRlncSig in the training cohort. The 46 lncRNAs with increased risk (hazard ratio > 1) were found to be associated with overall survival (*P* < 0.001) (**Figure 3A**). In addition, LASSO Cox regression analysis was used to obtain a risk model containing nine variables when the partial likelihood deviation reached the minimum, and the best log (lambda) was -3.1 (**Figures 3B, C**), and stepwise multivariate Cox regression analyses were performed to construct a risk model for HCC survival prediction. Five of the nine lncRNAs retained prognostic significance in these analyses and were used to construct the risk model (**Figures 3D** and **Table 2**). A co-expression network of these five independent lncRNAs and pyroptosis-related genes was established (**Figures 3E, F**).

**Table 2:** Multivariate cox regression analysis of five prognostic lncRNAs.

LncRNAs	Coefficient	HR	HR (95% CI)	*P* value
POLH-AS1	0.2608	1.2980	1.0285-1.6381	0.0280
LINC01224	0.1119	1.1184	0.9697-1.2899	0.1241
MKLN1-AS	0.2182	1.2438	1.0245-1.5101	0.0275
LINC02614	0.6288	1.8754	1.0597-3.3192	0.0309
MIR210HG	0.0291	1.0296	1.0003-1.0597	0.0479

**Figure 4:**
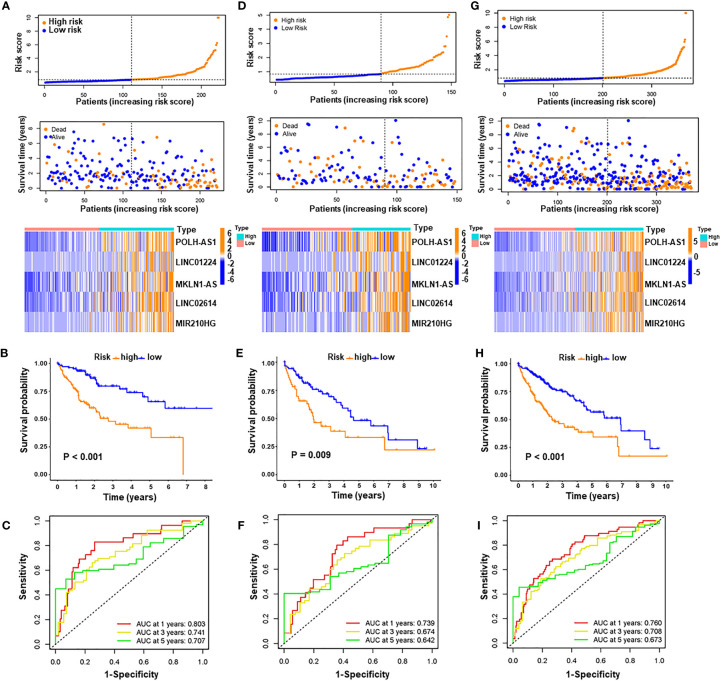
Evaluation and validation of pyroptosis-related lncRNA signature for overall survival in patients with HCC in three datasets. Risk scores and expression profiles of five-lncRNA signature in the high- and low-risk groups showed in the training cohort **(A)**, testing cohort **(D)**, and entire cohort **(G)**. Kaplan–Meier survival and ROC analyses in the training cohort **(B, C)**, testing cohort **(E, F)**, and entire cohort **(H, I)**, respectively.

A prognostic signature was constructed based on the expression levels of five pyroptosis-related lncRNAs, and the risk score was calculated as follows: risk score = 0.2608 × expression of POLH-AS1 + 0.1119 × expression of LINC01224 + 0.2182 × expression of MKLN1-AS + 0.6288 × expression of LINC02614 + 0.0291 × expression of MIR210HG. In the above formula, all five lncRNAs (POLH-AS1, LINC01224, MKLN1-AS, LINC02614, and MIR210HG) had positive coefficients, indicating that they were risk factors, and their overexpression was associated with poor prognosis. HCC patients with risk scores equal to or above the median PRlncSig score of 0.832 in the training cohort were classified as the high-risk group, and those with risk scores lower than the median were classified as the low-risk group (**Supplementary Table 6**). The classification ability of the risk signature was confirmed by PCA and t-SNE analysis (**Supplementary Figures 1A, B**). The patients in the high-risk group had higher expression of the five PRlncRNAs, and their overall survival time decreased as the risk score increased (**Figure 4A**), and the high-risk group had significantly shorter overall survival time than the low-risk group (**Figure 4B**). The same risk score formula was applied to the testing cohort and the entire cohort, and the results were similar to those obtained in the training cohort (**Supplementary Table 6** and **Figures 4D, E, G, H**). ROC curve analysis in the training cohort gave area under the curve (AUC) values for PRlncSig of 0.803, 0.741, and 0.707 for 1-, 3-, and 5-year survival, respectively (**Figure 4C**), and the corresponding AUC values were 0.739, 0.674, and 0.642 in the testing cohort (**Figure 4F**) and 0.760, 0.708, and 0.673 in the entire cohort (**Figure 4I**).

We used Xijing cohort as an external validation to test the validity and robustness of the PRlncRNAs. We performed qRT-PCR to measure the expression level of the five lncRNAs and the risk scores of every patient were calculated the formula of above risk score (**Supplementary Table 7**). And the distribution of the overall survival, risk score, and lncRNA expression pattern was shown in **Supplementary Figure 2A**. Consistent with bioinformatics analysis, our results indicated that the high-risk group had significantly shorter overall survival time than the low-risk group (**Supplementary Figure 2B**). Moreover, the AUCs were evaluated for 1-year (AUC = 0.722), 2-year (AUC = 0.781), 3-year (AUC = 0.686) survival (**Supplementary Figure 2C**). These results indicate that the risk model has good sensitivity and specificity in predicting the prognosis of HCC patients.

### Correlations Between PRlncSig and Clinicopathologic Characteristics in HCC

To examine whether PRlncSig was an independent prognostic factor in HCC, we performed univariate and multivariate Cox regression analyses on the three cohorts (training, testing, and entire) with respect to variables including age, gender, grade, stage, and PRlncSig. In the univariate Cox regression analysis, PRlncSig and tumor stage were significantly associated with overall survival of HCC patients in the training cohort (**Table 3**). Multivariate Cox regression analysis using these two variables revealed that PRlncSig was an independent prognostic factor for overall survival of HCC patients (**Table 3**). These results were verified in the testing and entire cohorts (**Table 3**).

**Table 3:** Univariate and multivariate cox regression analysis of PRLncSig and prognosis.

Clinicopathological characteristics	Univariable analysis			Multivariable analysis		
	HR	HR (95% CI)	*P* value	HR	HR (95% CI)	*P* value
**Training cohort (n =222)**						
Age	1.0065	0.9870-1.0264	0.5174			
Gender	1.5228	0.9066-2.5579	0.1119			
Grade	1.0517	0.7563-1.4624	0.7646			
Stage	2.2508	1.6750-3.0245	< 0.001	2.0908	1.5320-2.8533	< 0.001
PRLncSig	1.1888	1.1243-1.2570	< 0.001	1.1508	1.0846-1.2211	< 0.001
**Testing cohort (n = 148)**						
Age	1.0137	0.9919-1.0360	0.2191			
Gender	1.0282	0.5880-1.7981	0.9223			
Grade	1.2540	0.8360-1.8813	0.2740			
Stage	1.2836	0.9497-1.7350	0.1043			
PRLncSig	1.5943	1.1798-2.1546	0.0024	1.5930	1.1416-2.2228	0.0062
**Entire TCGA cohort (n = 370)**						
Age	1.0101	0.9956-1.0249	0.1736			
Gender	1.2892	0.8833-1.8817	0.1879			
Grade	1.1332	0.8813-1.4569	0.3296			
Stage	1.7136	1.3927-2.1084	< 0.001	1.6411	1.3232-2.0353	< 0.001
PRLncSig	1.1739	1.1195-1.2309	< 0.001	1.1451	1.0882-1.2049	< 0.001

Univariate analysis and Multivariate analysis, Cox proportional hazards regression model; HR, hazard ratio; 95% CI, 95% confidence interval.

**Figure 5:**
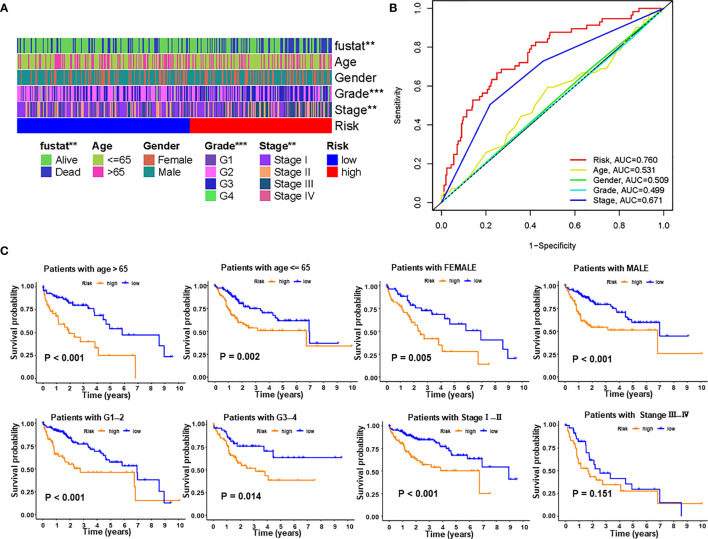
Correlations between risk score and different clinicopathological characteristics of HCC in the entire cohort. **(A)** Strip chart showing relationships between clinical characteristics and PRlncSig score. **(B)** ROC analysis of 3-year overall survival for multiple prognostic indicators of HCC samples. **(C)** Clinical stratification analysis of overall survival of patients with HCC in the high- and low-risk groups by age, gender, histological grade, and tumor stage. **P < 0.01, ***P < 0.001.

We next explored the correlations between PRlncSig and clinical pathological characteristics in the entire cohort and the heatmap showed that there were significant differences between high- and low-risk groups in survival status, histological grade, and tumor stage (**Figure 5A**). Furthermore, we used ROC curves to assess the accuracy of the risk model. The AUC values for the PRlncSig risk score and for age, gender, grade, and tumor stage were 0.760, 0.531, 0.509, 0.499, and 0.671, respectively (**Figure 5B**). Clinical stratification analysis was performed in the entire TCGA cohort according to the clinicopathologic factors such as age, gender, grade, and tumor stage; the results showed that patients in the high-risk group had significantly poorer prognosis than those in the low-risk group across all clinically stratified subgroups with the exception of tumor stage III–IV (**Figure 5C**). Together, these results indicate that the prognostic significance of PRlncSig in HCC patients is independent of other clinicopathologic features.

### Construction and Evaluation of the Prognostic Nomogram

**Figure 6:**
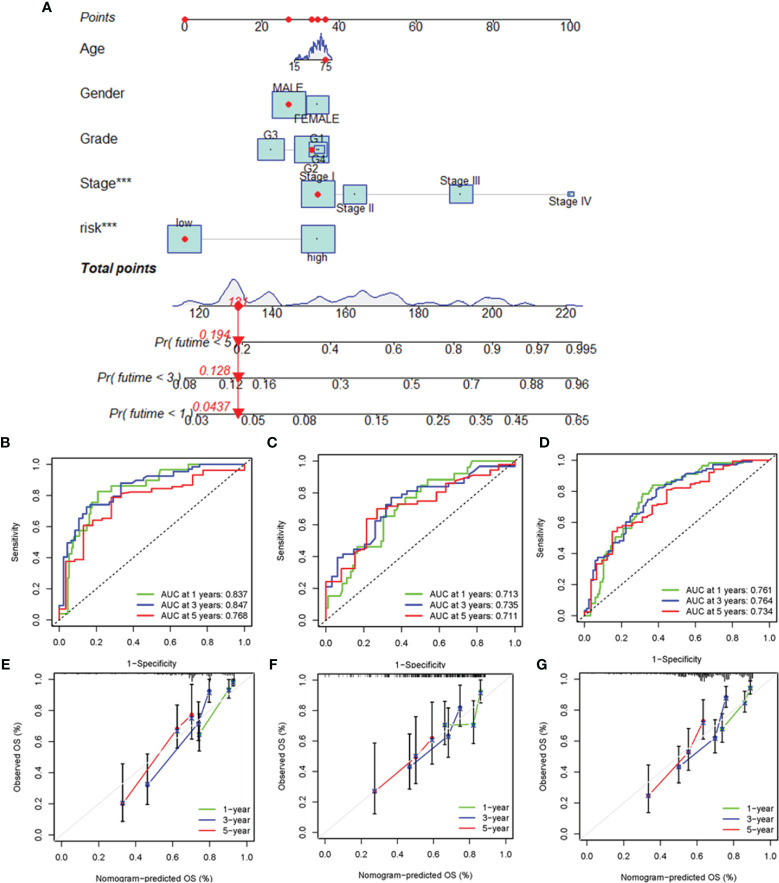
Construction and evaluation of the nomogram for clinicopathological characteristics and risk signature. **(A)** Nomogram combining PRlncSig and clinicopathological characteristics for predicting prognosis of HCC patients in the training cohort. ROC analysis of the predictions of 1-, 3-, and 5-year survival by the nomogram in the training cohort **(B)**, testing cohort **(C)**, and entire cohort **(D)**. Calibration curve analysis of the nomogram for the probability of overall survival at 1, 3, and 5 years in the training cohort **(E)**, testing cohort **(F)**, and entire cohort **(G)**. ***P < 0.001.

To enhance the clinical applicability of the risk model, we constructed a nomogram to predict the 1-, 3-, and 5-year overall survival probabilities of HCC patients in the training cohort based on various clinicopathologic factors, including PRlncSig risk score, age, gender, grade, and tumor stage (**Figure 6A**). The estimated AUC values of the ROC curves for the nomogram for 1-, 3-, and 5-year survival were 0.837, 0.847, and 0.768, respectively (**Figure 6B**). Similarly, the 1-, 3-, and 5-year AUCs were 0.719, 0.735, and 0.711 in the testing cohort (**Figure 6C**), and 0.761, 0.764, and 0.734 in the entire cohort, respectively (**Figure 6D**). The calibration curves of the nomogram revealed that the prediction of survival was closely associated with the actual 1-, 3-, and 5-year survival rates in the three cohorts (**Figures 6E–G**). Overall, these findings indicate that the nomogram represent an improved means of predicting the survival of HCC patients with advantages over the use of individual diagnostic characteristics.

### GSEA Analysis and Comparison of PRlncSig With Other Prognostic LncRNA-Related Signatures

**Figure 7:**
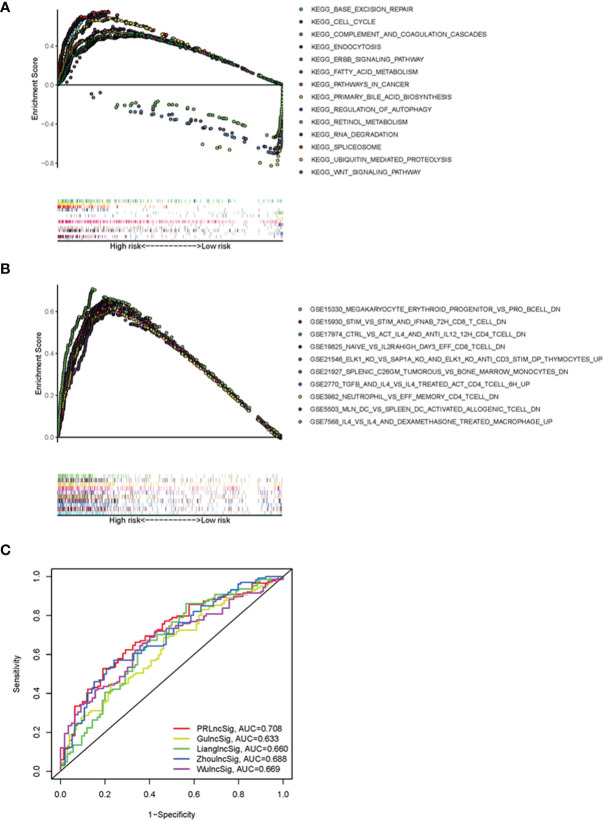
Functional GSEA analysis of PRlncSig and model comparisons. **(A)** Representative KEGG pathways significantly enriched in high-risk patients. **(B)** Immune-related signatures significantly enriched in high-risk patients. **(C)** ROC analysis of the prediction of 3-year survival by PRlncSig and four other signatures.

To elucidate the underlying molecular mechanisms involving PRlncSig in HCC, we performed GSEA to identify PRlncSig-mediated signaling pathway. RNA degradation (normalized enrichment score (NES) = 2.15, FDR = 0.001), ubiquitin-mediated proteolysis (NES = 2.03, FDR = 0.011), cell cycle (NES = 2.00, FDR = 0.014), WNT signaling pathway (NES = 1.75, FDR = 0.030), and pathways in cancer (NES = 1.74, FDR = 0.031) were significantly enriched in the high-risk group (**Figure 7A**), whereas complement and coagulation cascades (NES = -2.03, FDR = 0.003), primary bile acid biosynthesis (NES = -1.90, FDR = 0.019), and fatty acid metabolism (NES = -1.65, FDR = 0.153) were significantly enriched in the low-risk group (**Figure 7A**). The GSEA analysis also showed that high risk scores based on PRlncSig were significantly associated with numerous immune processes, including GSE19825_NAIVE_VS_IL2RAHIGH_DAY3_EFF_CD8_TCELL_DN (NES = 2.18, FDR = 0.001), GSE7568_IL4_VS_IL4_AND_DEXAMETHASONE_TREATED_MACROPHAGE_UP (NES = 2.14, FDR = 0.002), GSE21927_SPLENIC_C26GM_TUMOROUS_VS_BONE_MARROW_MONOCYTES_DN (NES = 2.13, FDR = 0.001), and GSE5503_MLN_DC_VS_SPLEEN_DC_ACTIVATED_ALLOGENIC_TCELL_DN (NES = 2.12, FDR = 0.001) (**Figure 7B**). These results indicate that PRlncSig may be associated with the tumor immune microenvironment.

We further compared the prediction efficiency of the PRlncSig with that of three previously published prognostic signatures, the lncRNA signatures derived from Zhou’s study (ZhoulncSig) (22), Gu’s study (GulncSig) (23), Liang’s study (LianglncSig) (24), Wu’study (WulncSig) (25), using the same TCGA_LIHC dataset. As shown in **Figure 7C**, the AUC of the 3-year overall survival curve for PRlncSig was 0.708, higher than those obtained for GulncSig (AUC = 0.633), ZhoulncSig (AUC = 0.688), LianglncSig (AUC = 0.660), WulncSig (AUC = 0.669). These results demonstrate the superiority of our signature for prognostic prediction in HCC patients.

### Associations of PRlncSig Model With Immune Cell Infiltration and Therapeutic Effect

**Figure 8:**
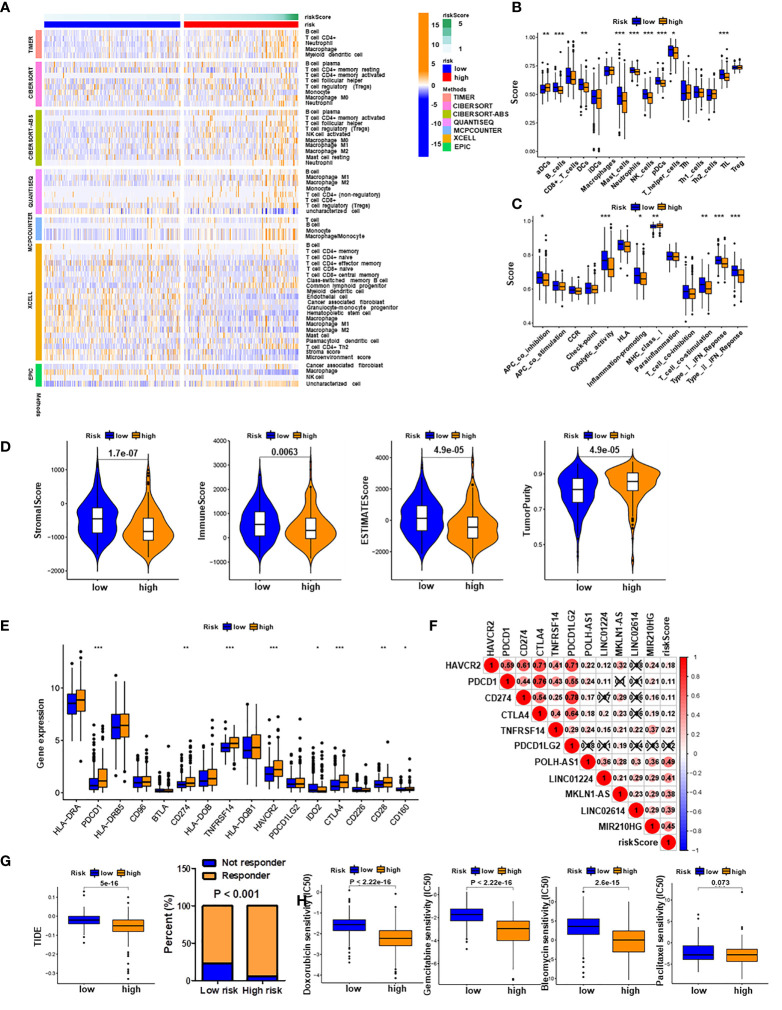
Immune infiltration analysis and prediction of immunotherapy and chemotherapy response. **(A)** Heatmap of all significantly differential immune responses between high- and low-risk groups based on TIMER, CIBERSORT, CIBERSORT-ABS, QUANTISEQ, MCPCOUNTER, XCELL, and EPIC algorithms. Immune cell scores **(B)** and immune function scores **(C)** in high- and low-risk groups based on ssGSEA algorithm. **(D)** Comparison of stromal scores, immune scores, estimate scores, and tumor purity between HCC patients in high- and low-risk groups. **(E)** Expression levels of immune checkpoint genes in high- and low-risk groups. **(F)** Associations of PRlncSig score with pyroptosis-related lncRNAs and immune checkpoint genes. **(G)** Comparison of TIDE prediction scores (left) and response to immunotherapy (right) between the high- and low-risk groups in the TCGA_LIHC dataset. **(H)** Assessment of sensitivity to several chemotherapeutics (doxorubicin, gemcitabine, bleomycin, and paclitaxel in the high- and low-risk groups. *P < 0.05, **P < 0.01, ***P < 0.001.

To investigate the relationship between the PRlncSig model and tumor-infiltrating immune cells, we used the TIMER, CIBERSORT, QUANTISEQ, MCPCOUNTER, XCELL, and EPIC algorithms to estimate the differences in immune infiltration between the low-risk and high-risk groups. A heatmap of all significantly differential immune responses is shown in **Figure 8A**. Comparative analyses of immune cell subpopulations revealed significant differences in the infiltrating levels of immune cells (including aDCs, B cells, DCs, mast cells, neutrophils, NK cells, pDCs, T helper cells, and TIL) and immune function (APC co-inhibition, cytolytic activity, inflammation promoting, MHC class I, T cell co-stimulation, type I IFN response and type II IFN response) between the low-risk and high-risk group (**Figures 8B, C**). We also found that the infiltrating levels of B cells, NK cells, and pDCs cells were negatively correlated with risk score, as were immune function, type II IFN response, cytolytic activity and T cell co-stimulation (**Supplementary Figures 3A, B**). Moreover, the correlations between single lncRNAs in the PRlncSig and tumor-infiltrating immune cells were further explored using the CIBERSORT algorithm (**Supplementary Figure 3C**), and the results showed that LINC01224 had significant positive correlations with T follicular helper cells, memory-activated CD4 T cells, and resting dendritic cells, whereas it showed a significant negative correlation with activated NK cells. MIR210HG had significant negative correlations with regulatory T cells, resting dendritic cells, and naïve B cells; and POLH-AS1 had a significant positive correlation with T follicular helper cells. Next, we analyzed the tumor microenvironment (TME) of each sample and related characteristics, including StromalScore, ImmuneScore, EstimateScore, and TumorPurity, in the two groups. The results showed that HCC patients in the high-risk group had lower stromal, immune, and estimate Scores but higher tumor purity scores compared with those in the low-risk group (**Figure 8D** and **Supplementary Figure 4**).

Given the importance of checkpoint-based immunotherapy, we investigated the expression profiles of immune checkpoint genes in the two groups and found that several genes (HAVCR2, PDCD1, CD274, CTLA4, TNFRSF14, and CD28) were highly expressed in the high-risk group (**Figure 8E**). Subsequently, we examined the relationships between immune checkpoint genes and PRlncSig and found that five immune checkpoint genes, HAVCR2, PDCD1, CD274, CTLA4, and TNFRSF14, were positively correlated with PRlncSig risk score (*P* < 0.05; **Figure 8F**). Furthermore, the TIDE algorithm, which was developed to predict immunotherapy response, was used to calculate whether PRlncSig could predict immunotherapeutic benefit in the two groups. The patients in the high-risk group had significantly lower TIDE scores than those in the low-risk group, indicating that high-risk patients would show better responses to immunotherapy (**Figure 8G**). We also determined the IC50 values of four common chemotherapeutic drugs (doxorubicin, gemcitabine, bleomycin, and paclitaxel) in high-risk and low-risk patients; the high-risk group had lower IC50 values for doxorubicin (*P* < 2.22E-16), gemcitabine (*P* < 2.22E-16), and bleomycin (*P* < 2.6E-15) (**Figure 8H**). This may indicate that high-risk patients were more sensitive to these three drugs. There was no significant difference between the groups for paclitaxel (*P* = 0.073) (**Figure 8H**). In summary, these results indicate that PRlncSig is correlated with tumor-infiltrating immune cells in HCC to a certain extent, and that patients with high risk scores may show more positive responses to immunotherapy.

### Validation of Expression of LncRNAs in HCC Samples

**Figure 9:**
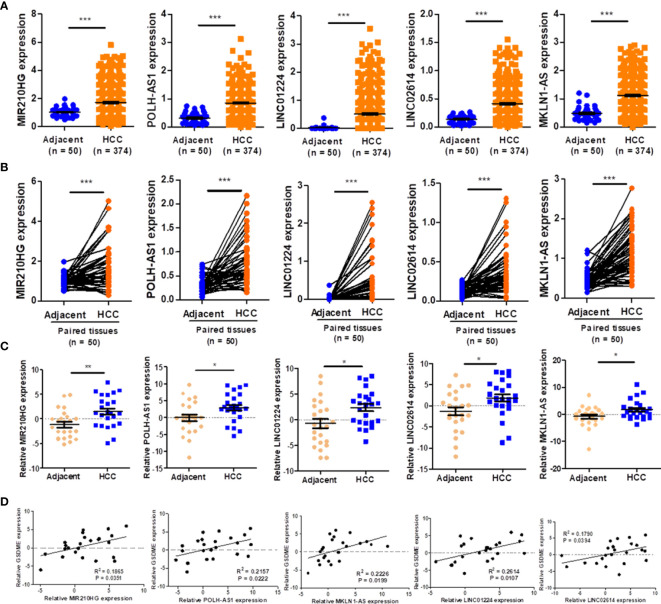
Validation of the expression of five lncRNAs. **(A)** The expression level of five lncRNAs in HCC and adjacent tissues, as indicated by TCGA_LIHC dataset. **(B)** Five lncRNAs in HCC and paired adjacent tissues according to the TCGA_LIHC dataset. **(C)** The expression of five lncRNAs in twenty-four pairs of HCC tissues and adjacent tissues verified using qRT-PCR (n = 24). **(D)** Associations of pyroptosis-related gene, GSDME and five lncRNAs expression levels verified using qRT-PCR (n = 24). *P < 0.05, **P < 0.01, ***P < 0.001.

As demonstrated in **Figure 9A**, the expression of five lncRNAs (POLH-AS1, LINC01224, MKLN1-AS, LINC02614, and MIR210HG) were significantly upregulated in HCC tissues compared with paired adjacent tissues. Similarly, the expression of five lncRNAs was respectively upregulated in HCC tissues in comparison with the paired adjacent tissues (**Figure 9B**). Furthermore, the results of expressions of five lncRNAs were further verified in 24 HCC tissues and adjacent tissues by qRT-PCR analysis (**Figure 9C**). We found that the expression levels of eight representative pyroptosis-related genes (GSDMA, GSDMB, GSDME, CASP1, CASP4, IL1B, IL18 and CASP3) were highly expressed in the high-risk group (**Supplementary Figure 5A**). The expressions of representative pyroptosis-related genes were positively correlated with PRlncSig risk score except GSDMD gene and the expression level of GSDME gene also showed high positively correlations with five lncRNAs (**Supplementary Figure 5B**). The qRT-PCR results were consistent with those of the bioinformatics analysis (**Figure 9D**). The results indicated that the risk-related roles of the five lncRNAs in HCC.

## Discussion

The pathogenesis of HCC is very complicated, involving transcriptional misregulation and cell cycle disorder (26, 27), somatic mutation (27) and abnormal pyroptosis (28). LncRNAs can affect tumorigenesis and tumor progression in a variety of ways, including regulation of cell proliferation and apoptosis (29), influencing drug sensitivity (30), and regulation of cell pyroptosis (31). Various prognostic prediction models for HCC patients based on lncRNA signatures have been reported (22, 32, 33). However, owing to the tumor heterogeneity of HCC and the different analysis strategies used in each study, the lncRNA signatures identified are different and it is necessary to develop individualized treatment strategies and improve the precision of prediction (4). Pyroptosis, a newly recognized type of programmed cell death, may have an inhibitory role in the progression of HCC (8, 34). However, the lncRNAs involved in the regulation of pyroptosis and the potential of pyroptosis-related lncRNA signatures to predict prognosis of HCC patients have still remained unclear. In this study, we constructed a prognostic signature of five pyroptosis-related lncRNAs and showed that it could precisely distinguish between high- and low-risk patients. This PRlncSig could thus serve as a prognostic risk model to predict the survival of HCC patients. ROC curves were constructed and used to show that the PRlncSig model had moderate predictive ability on three datasets (the entire TCGA cohort and two subgroups). In addition, our newly developed nomogram model could visually predict the 1-, 3- and 5-year survival of individual HCC patients and could be used to improve clinical decision-making. So far, Wu et al. showed that nine pyroptosis-related lncRNA signature provided a prediction for the prognosis of HCC patients (25). Of note, the prediction efficiency of PRlncSig was superior to those of clinical characteristics. The prediction accuracy of our PRlncSig also exceeded those of four recently reported lncRNA prediction signatures for HCC (22–25).

We identified five lncRNAs, POLH-AS1, LINC01224, MKLN1-AS, LINC02614, and MIR210HG, that were highly expressed in the high-risk group and associated with poor prognosis. Previous studies have suggested that LINC01224 promotes HCC progression by upregulating the expression of CHEK1 (35), as well as accelerating cell proliferation and invasion in non-small-cell lung cancer (36). Gao et al. demonstrated that knockdown of MKLN1-AS expression prevented cell proliferation of HCC (37), and Wang et al. showed that MIR210HG was an oncogenic lncRNA in HCC, whose overexpression predicted poor prognosis (38). We identified two new risk-related lncRNAs (POLH-AS1 and LINC02614) in HCC that had not previously been reported in the literature. Our data showed that the expression level of LINC02614 was positively correlated with that of CASP8, whereas the expression level of POLH-AS1 was significantly correlated with those of ten pyroptosis-related genes (BAK1, SCAF11, BAX, PLCG1, CHMP4B, CASP6, CHMP3, HMGB1, CASP3, and TP53) (**Supplementary Table 5** and **Figures 3E, F**). Further studies are needed to confirm the effects of POLH-AS1 and LINC02614 in the development of HCC *via* a pyroptosis mechanism.

We investigated the underlying molecular mechanism by which PRlncSig is involved in HCC through GSEA analysis, and the results showed that ubiquitin-mediated proteolysis, cell cycle, WNT signaling pathway, and pathways in cancer may have important roles in the poor prognosis of HCC patients in the high-risk group. Previous research showed that lncRNA-Fendrr inhibited the ubiquitination and degradation of NLRC4 through E3 ubiquitin ligase HERC2 to regulate the pyroptosis of microglia (39). Knockdown of LINC01224 could block cell cycle progression of HCC (35). Silencing MIR210HG inhibited WNT signaling pathways by targeting the miR-337-3p/137-HMGA2 regulatory axis (40). In addition, we confirmed that high PRlncSig risk score was correlated with numerous immune processes. Shao et al. reported that pyroptosis-related genes were related to prognosis and immune microenvironment infiltration in gastric cancer (16). Zhang et al. found that lncRNA Neat1 stabilized mature caspase-1 to promote inflammasome stimulation and trigger pyroptosis (41). As such, it will be interesting to further investigate the vital interactions between the PRlncSig and tumor immunoregulation during the progression of HCC.

The immune microenvironment of HCC can be characterized by the ImmuneScore, StromalScore, immune checkpoints, and the proportions and functions of immune cell subpopulations. Tumor cells rely on the immune suppression of the host to escape immune surveillance, thereby promoting tumor survival and progression (42, 43). This may result in decreased the infiltrating levels of immunoreactive cells (such as M1 macrophages and helper T cells) and increased immunosuppressive cells (such as M2 macrophages and regulatory T cells). Our analysis found that HCC patients in the high-risk group had lower infiltrating levels of helper T cells, mast cells, and pDCs cells than low-risk patients. The results also indicated that the poor prognosis of high-risk patients could be ascribed to lower immunoreactivity in the TME. In addition, the HCC patients in the high-risk group had lower stromal scores and immune scores but higher tumor purity compared with those in the low-risk group, suggesting that PRlncSig could be regarded as a novel immune indicator in HCC. Moreover, immune checkpoint inhibitors have improved clinical decision-making in cancer treatment (44, 45). Zhang et al. found that glioma patients with high risk had higher expression of immune checkpoint genes, including PDCD1LG2, TNFRSF14, and PDCD1 (46). Wang et al. found increased expression of CD274, CTLA4, HAVCR2, and TIGIT in a high-risk group of HCC patients (47). Consistent with previous studies, PRlncSig score was positively correlated with the expression levels of immune checkpoint genes (HAVCR2, PDCD1, CD274, CTLA4, and TNFRSF14). In addition, HCC patients in the high-risk group had significantly lower TIDE scores, indicating better responses to immunotherapy. Therefore, our PRlncSig model could contribute to personalized treatment by enabling clinicians to judge a patient’s likely response to immunotherapy.

Although PRlncSig shows promise, the current study had several limitations. First, our signature was validated mainly using the TCGA database, and external validation of the PRlncSig using different databases and large-scale multicenter cohorts is necessary. Second, further experimental studies are required to elucidate the role of the five predictive lncRNAs in HCC.

## Conclusions

In summary, we constructed a pyroptosis-related lncRNA signature, PRlncSig, to predict the prognosis of HCC patients. Further analysis indicated that PRlncSig score could serve as an independent prognostic indicator and might be associated with tumor immune infiltration levels and even the efficacy of tumor immunotherapy.

## Data Availability Statement

The original contributions presented in the study are included in the article/**Supplementary Material**, further inquiries can be directed to the corresponding authors.

## Ethics Statement

The studies involving human participants were reviewed and approved by the Ethical Committee and Institutional Review Board of Fourth Military Medical University. The patients/participants provided their written informed consent to participate in this study. Written informed consent was obtained from the individual(s) for the publication of any potentially identifiable images or data included in this article.

## Author Contributions

Z-KL, K-FW, and R-YZ were involved in the data analyses, carried out the experiments, wrote, reviewed, and edited the manuscript. L-MK, R-ZS, J-JL, and CL contributed to prepare figures, data analyses and reviewed the manuscript. ML and Y-LY prepared figures and data analyses. CZ, N-SZ, and Y-HL contributed to the discussion, and reviewed the manuscript. Z-KL, Z-NC, HB, and DW conceived the study, designed and oversaw the study, evaluated data and revised the manuscript. All authors contributed to the article and approved the submitted version.

## Funding

This work was supported by the National Natural Science Foundation of China (82130084, 81874155, 81872482 and 82002940).

## Conflict of Interest

The authors declare that the research was conducted in the absence of any commercial or financial relationships that could be construed as a potential conflict of interest.

## Publisher’s Note

All claims expressed in this article are solely those of the authors and do not necessarily represent those of their affiliated organizations, or those of the publisher, the editors and the reviewers. Any product that may be evaluated in this article, or claim that may be made by its manufacturer, is not guaranteed or endorsed by the publisher.

## Abbreviations

HCC, hepatocellular carcinoma; lncRNAs, long non-coding RNAs; PRlncSig, pyroptosis-related lncRNA signature; TCGA, The Cancer Genome Atlas; RNA-seq, RNA sequencing; FPKM, fragments per kilobase million; TPM, transcripts per million; DEGs, differentially expressed genes; FDR, false discovery rate; MSigDB, molecular signatures database; LASSO, least absolute shrinkage and selection operator; ROC, receiver operating characteristic; PCA, principal components analysis; t-SNE, t-distributed stochastic neighbour embedding; ssGSEA, single-sample gene set enrichment analysis; TIDE, tumor immune dysfunction and exclusion; GO, gene ontology; KEGG, Kyoto Encyclopedia of Genes and Genomes.
