# Supplementary material for: Pyroptosis-Related LncRNA Signature Predicts Prognosis and Is Associated With Immune Infiltration in Hepatocellular Carcinoma
Source: Front Oncol. 2022 Mar 3;12:794034. doi: 10.3389/fonc.2022.794034 (PMC8927701; doi:10.3389/fonc.2022.794034)
Supplement: Supplementary file 1 [file DataSheet_1.docx]

Supplementary Material

# Supplementary Figures and Tables

## Supplementary Figures

**Supplementary Figure 1. PCA and t-SNE analysis.** PCA (**A**) and t-SNE analysis (**B**) between high- and low-risk groups in the training cohort, testing cohort, and entire cohort, respectively.

**
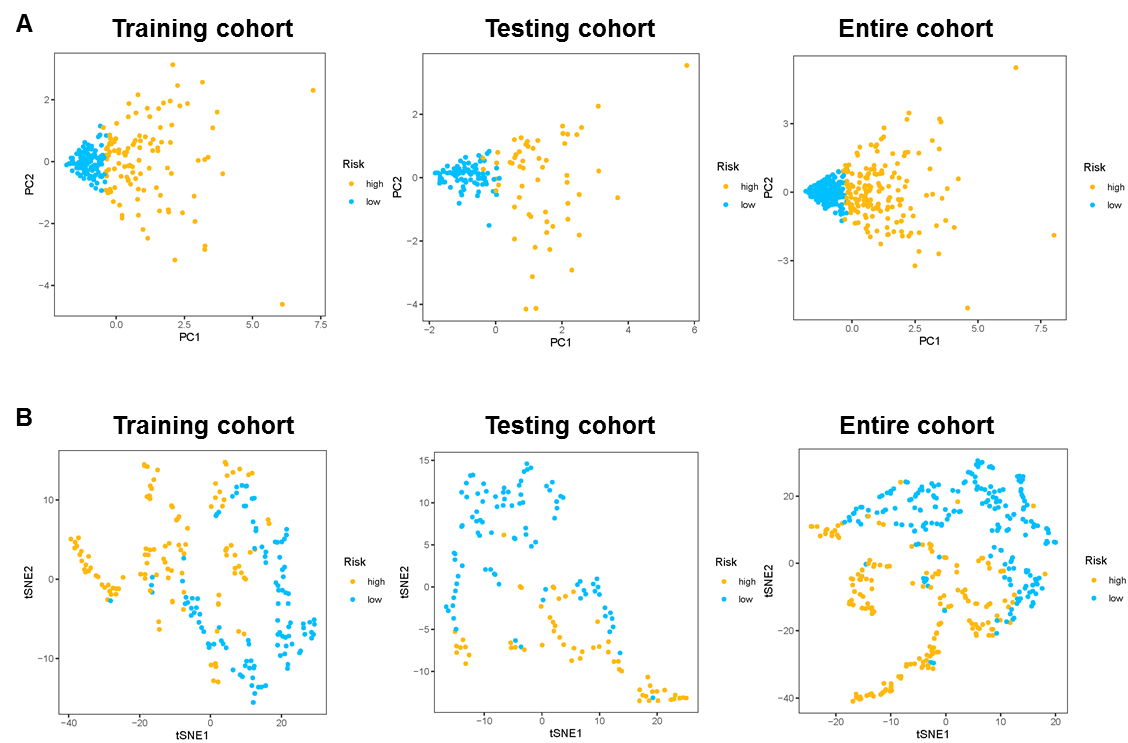
**

**Supplement Figure 2. Validation of pyroptosis-related lncRNA signature for overall survival in patients with HCC in the internal cohort.** (A)The expression profiles of five-lncRNA signature in the high- and low-risk groups. (B)Kaplan–Meier survival of patients with HCC based on the prognostic signature. (C)ROC analyses of 1-, 2-, and 3-year overall survival predicted by the prognostic signature.


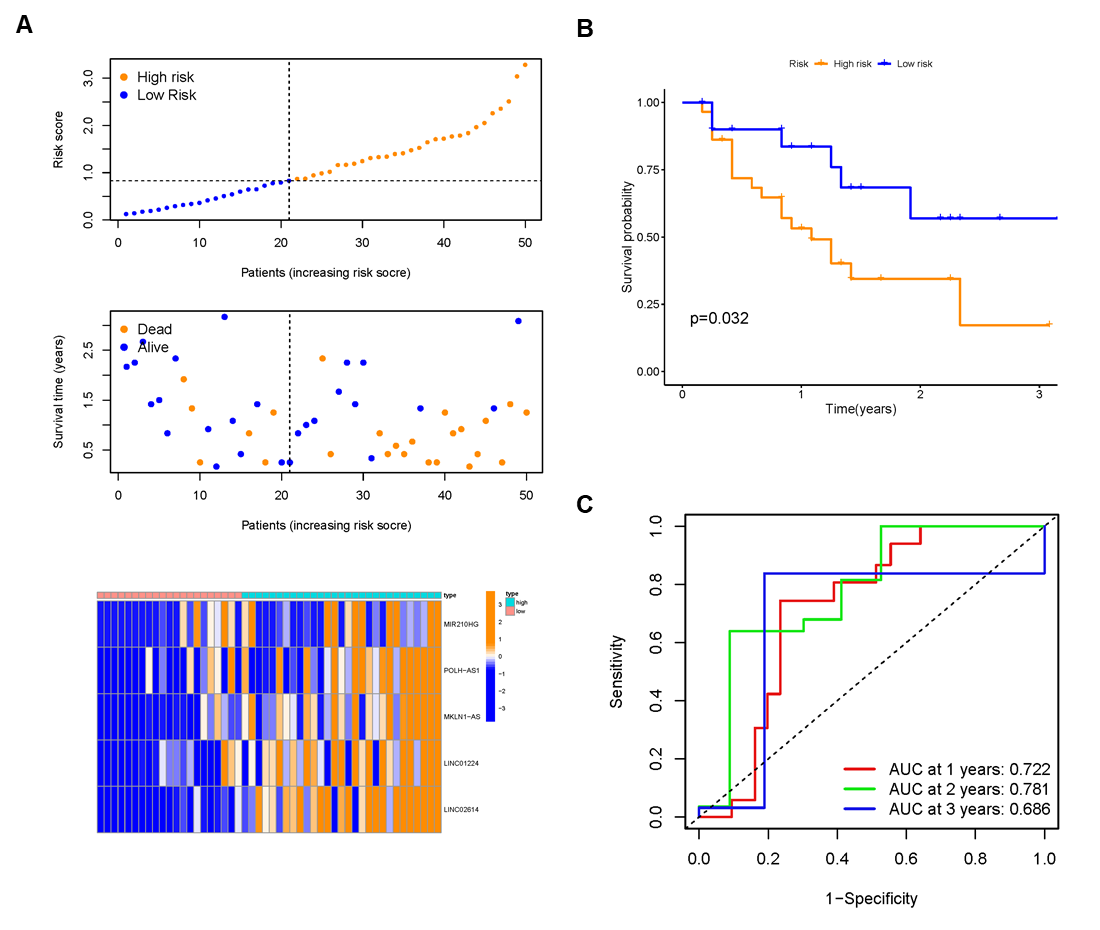


**Supplementary Figure 3. Correlation analysis of PRlncSig score and immune cell infiltration.** Correlations of PRlncSig score with immune cell scores (**A**) and immune function scores (**B**) in the TCGA_LIHC dataset. (C) Heatmap showing the correlations between the five pyroptosis-related lncRNAs and immune cell infiltration. Red indicates positive correlation, and blue indicates negative correlation. **P* < 0.05, ***P* < 0.01.

**
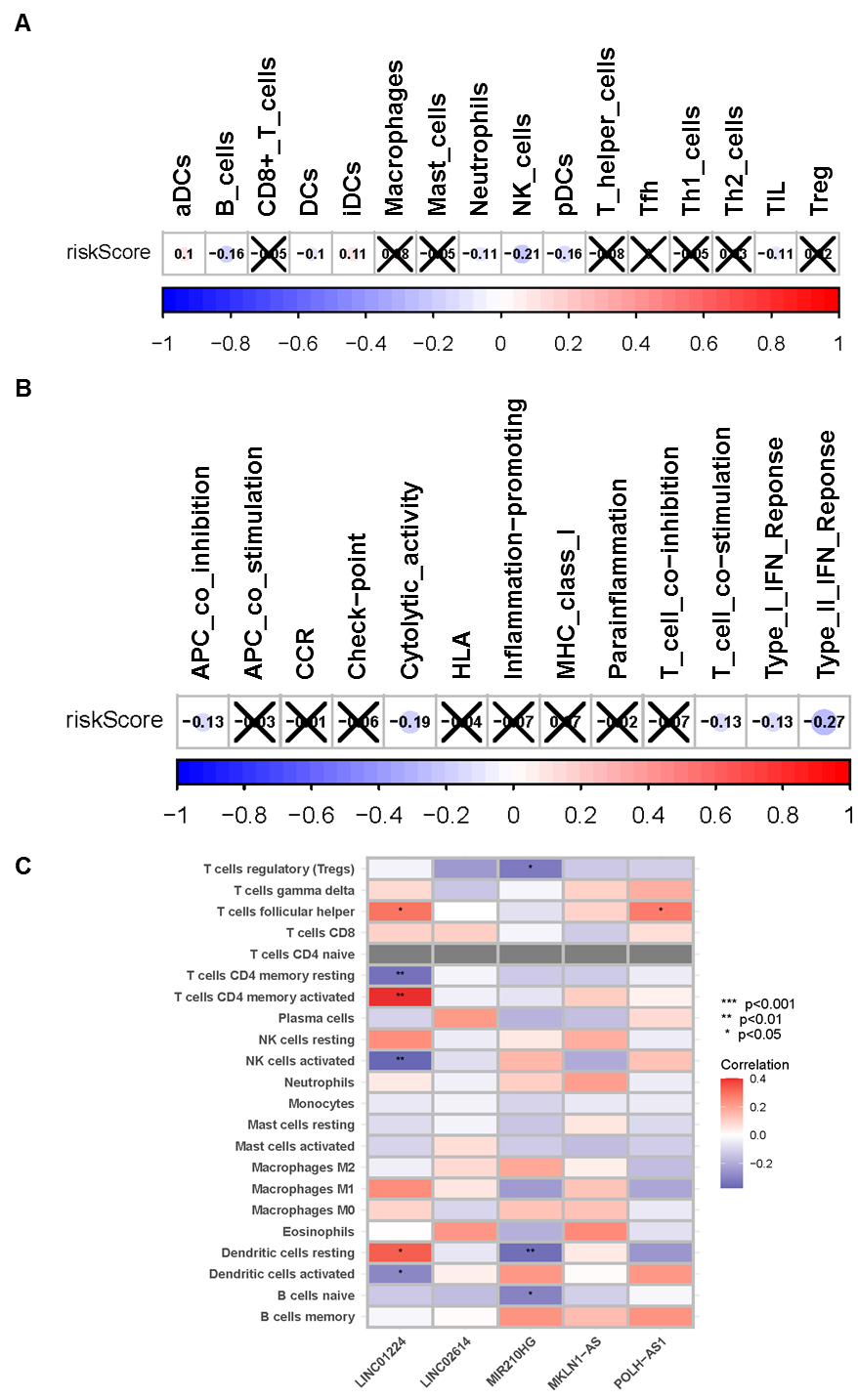
**

**Supplementary Figure 4. Heatmap of immune microenvironment landscape for each sample.** Immune microenvironment analysis using ESTIMATE algorithm in high- and low-risk patients.


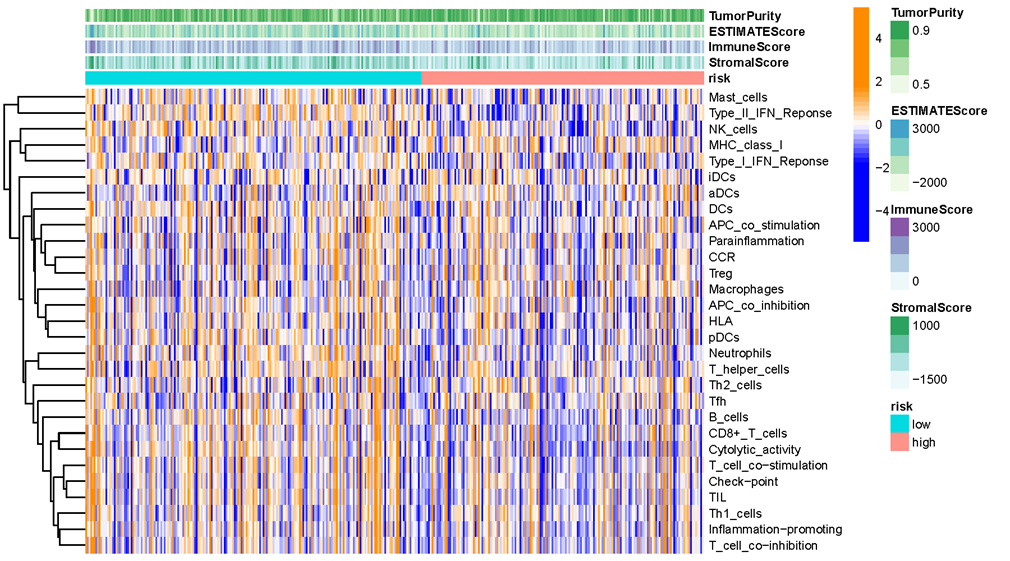


**Supplementary Figure 5.** **Correlation analysis of PRlncSig score and pyroptosis-related genes.**

(A) Expression levels of pyroptosis-related genes in high- and low-risk groups. (**B**) Associations of pyroptosis-related genes and five lncRNAs expression levels.


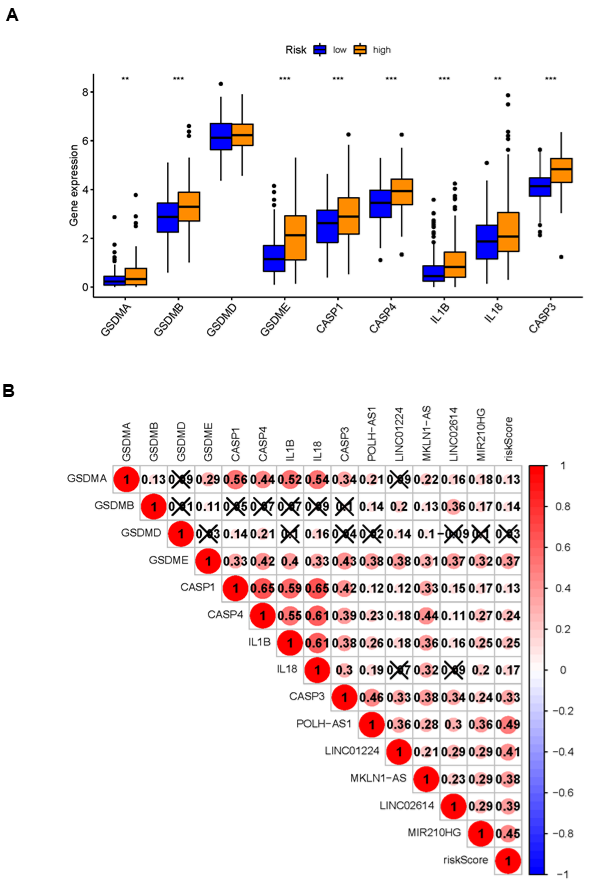


## Supplementary Tables

**Supplementary Table S1. Pyroptosis-related genes.**

**Supplementary Table S2. Markers of immune cells and pathways.**

**Supplementary Table S3. Primers for real-time PCR.**

**Supplementary Table S4. Differentially expressed pyroptosis-related genes and lncRNAs.**

**Supplementary Table S5. Correlation analysis between pyroptosis-related genes and lncRNAs.**

**Supplementary Table S6. Classification of risk groups in each cohort based on HCC patients’ risk scores.**

**Supplementary Table S7. Classification of risk groups in external cohort based on the HCC patients’ risk scores.**
