# Supplementary material for: Pyroptosis-Related LncRNA Signature Predicts Prognosis and Is Associated With Immune Infiltration in Hepatocellular Carcinoma
Source: Front Oncol. 2022 Mar 3;12:794034. doi: 10.3389/fonc.2022.794034 (PMC8927701; doi:10.3389/fonc.2022.794034)
Supplement: Supplementary file 4 [file Table_3.docx]

Supplementary Table S3. Primers for real-time PCR.

| Gene | Forward primer | Reverse primer |
| --- | --- | --- |
| MKLN1-AS | AAAGAGTATGTCGCTTATTGTCTAAGA | ATCCTGCTGACTTACTCCAGATGT |
| MIR210HG | GCAGGCACAGGTGTGGTCATATC | AGGCAGGCTCAGCAGACAGG |
| LINC02614 | CTAGGAGAAGGCAGGAGAGAGA | CCAAGCCAATAGTCCAGCCAA |
| POLH-AS1 | GGCAGGTGGATCATTTGAGGTCAG | CCAAGTAGCTGGGATTACAGGCATG |
| LINC01224  GSDME | AGAGCTTGGGATCGCTTTCTG  CACACTGTGCCACTTGCTTC | TTACTCAGGTGCCTTTCCCAC  GTCAGCTGAGGCAAACAAGC |
| GAPDH | CGGAGTCAACGGATTTGGTCGTAT | AGCCTTCTCCATGGTGGTGAAGAC |
